# Fine details in complex environments: the power of cryo-electron tomography

**DOI:** 10.1042/BST20170351

**Published:** 2018-06-22

**Authors:** Joshua Hutchings, Giulia Zanetti

**Affiliations:** Institute of Structural and Molecular Biology, Birkbeck College, Malet St., London WC1E 7HX, U.K.

**Keywords:** cryo-electron microscopy, cryo-electron tomography, subtomogram averaging

## Abstract

Cryo-electron tomography (CET) is uniquely suited to obtain structural information from a wide range of biological scales, integrating and bridging knowledge from molecules to cells. In particular, CET can be used to visualise molecular structures in their native environment. Depending on the experiment, a varying degree of resolutions can be achieved, with the first near-atomic molecular structures becoming recently available. The power of CET has increased significantly in the last 5 years, in parallel with improvements in cryo-EM hardware and software that have also benefited single-particle reconstruction techniques. In this review, we cover the typical CET pipeline, starting from sample preparation, to data collection and processing, and highlight in particular the recent developments that support structural biology *in situ*. We provide some examples that highlight the importance of structure determination of molecules embedded within their native environment, and propose future directions to improve CET performance and accessibility.

## Introduction

**Figure 1.**
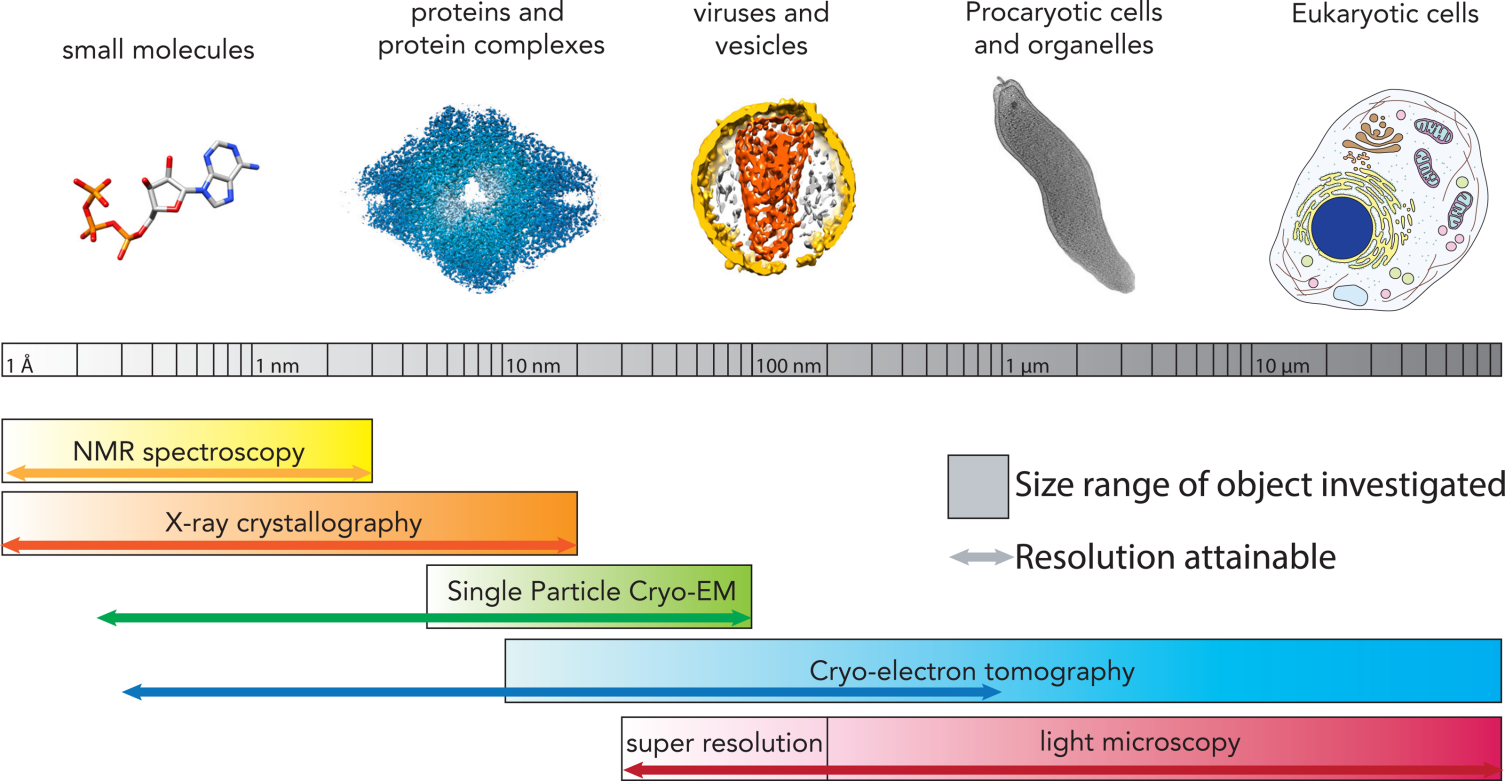
An overview of structural biology techniques and the biological objects they investigate. The size range of biological objects that can be studied is represented with thick bars, while arrows of corresponding colours indicate the resolution ranges that can be targeted. CET is ideally placed to resolve biological molecules at subnanometer resolutions, while studying large and complex assemblies such as eukaryotic cells. ATP is used as an example of small molecules, EMD-2984 was used as representative protein, a segmented tomogram from Mattei et al. [6] was used as representative of virus, EMD-2754 was used to extract a *Campylobacter jejuni* cell to represent bacteria.

The electron microscope provides a powerful tool to understand biological processes over a wide range of scales, from the determination of molecular structures to the characterisation of cell morphology (Figure 1).

For decades, electron microscopy (EM) of stained and sectioned cells helped define cell morphology and ultrastructure, understand the function of organelles, and identify the aetiology of many diseases. The introduction in the 1980s of cellular electron tomography [1] has brought morphological studies to a higher level, with 3D views resolving events through the depth of the cell. Great advances have followed upon the development of techniques to prepare cells for visualisation in cryo-conditions, including CEMOVIS (cryo-EM of vitrified specimens) [2] and more recently focused ion beam (FIB)–scanning electron microscopy (SEM) [3–5], which allow preservation of the native cellular structures. Cellular cryo-tomography can now yield reconstructions that are virtually free from deformations, achieving resolutions of better than 10 nm.

On a smaller scale, single-particle cryo-EM has become a very popular technique for protein structure determination. Thanks to recent hardware and software advances, current standards of single-particle reconstruction yield maps at near-atomic resolutions, comparable to those obtained with more ‘traditional' structural techniques. Single-particle cryo-EM is especially powerful because biological molecules are visualised in near-native conditions, and several conformational states can be present in the same preparation. A limitation of single-particle cryo-EM, as with all structural techniques, is that proteins are extracted away from their environment in the process of purification. Great biochemical efforts enable the purification of highly complex assemblies, which preserve many of the inherent biological interactions. Yet, information on the macromolecule's native context and its transient interactions cannot be recovered. For example, macromolecules embedded in a membrane or within larger pleomorphic assemblies are not generally amenable to single-particle cryo-EM, leaving a gap of structural information between molecules and cells.

Cryo-electron tomography (CET) and subtomogram averaging (STA) are fast-developing techniques that allow structure determination *in situ*, bridging the gap across biological scales. Visualisation of protein complexes to atomic detail, at work in their biological environment, will profoundly advance our understanding of molecular mechanisms. To date, a limited number of studies have been published, in which CET and STA have contributed insights into complex assemblies at progressively increasing resolutions, together with information on their physiological context [6–14]. Most studies so far have focused on assemblies that present a regular pattern, are large and recognisable, or are amenable to *in vitro* reconstitution, making structure determination by STA an achievable goal.

The recent and ongoing developments in sample preparation, data collection, and image processing are making molecular resolution achievable for progressively smaller, sparser, and more interconnected complexes *in situ*. Here, we review these recent advances that have underpinned the improvements in resolution and interpretability of cryo-tomograms. We provide a gallery of examples that demonstrate the potential of this approach for *in situ* structure determination.

## Sample preparation

**Figure 2.**
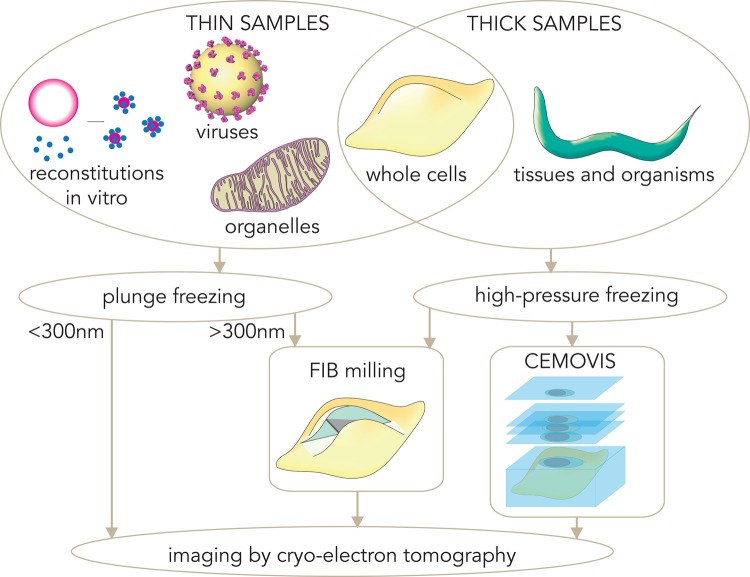
An overview of sample preparation approaches for CET. Freezing techniques must be adapted to sample thickness. Specimens thinner than a few microns can be plunge-frozen in liquid ethane, while thicker specimens must be frozen under high pressure to achieve vitrification. Whole cells can be plunge-frozen if they are very thin (like some bacterial cells), or if the aim is tomography of the thinnest peripheral regions. Thicker plunge-frozen material may need to be thinned to become electron-transparent in order to be imaged. High-pressure frozen blocks can be sectioned, although more recently FIB-milling has been successfully used to thin FIB-milled specimens.

Central to cryo-EM is the preservation of biological specimens in vitreous ice [15]. Plunge-freezing into liquid ethane is the method of choice to vitrify thin samples. Typical cryo-tomography samples that can be plunge-frozen remain confined to specimens such as reconstituted systems [8,16], viruses [7,11], isolated organelles [10,17], some bacterial cells [18], and peripheral regions of eukaryotic cells [19] (Figure 2).

If the plunge-frozen specimen is thinner than ∼300 nm, then it is possible to image it directly under the electron beam. Thicker specimens (for example, eukaryotic cell nuclear peripheries) require thinning for the electron beam to effectively penetrate [20]. This can be achieved by on-grid FIB-milling, which uses a focused beam of Gallium ions (Ga^+^) to mill away regions of cells that would normally be too thick for EM imaging, leaving a thin electrotransparent slab without devitrifying the specimen [3,4,21] (Figure 2). The process is carried out in a dual FIB/SEM microscope while under vacuum, to simultaneously monitor the FIB-milling process by SEM [5,22,23]. The milled specimen is then transferred to the cryo-TEM for data collection. Recent developments in correlative light and cryo-electron microscopy for FIB-milled specimens allow specific cellular processes to be targeted for tomography [24].

For specimens thicker than several microns, such as nuclear regions, tissues, and whole organisms (e.g. *Caenorhabditis elegans*), plunge-freezing is not sufficient to achieve the cooling rates needed for full vitrification, and high-pressure freezing (HPF) is necessary to avoid the formation of crystalline ice [25] (Figure 2). As for thicker plunge-frozen specimens, following HPF the vitrified samples must be thinned: cryo-ultramicrotomy has been used for ∼15 years to obtain sections with <100 nm depth for CEMOVIS [2,26] (Figure 2). More recently, FIB-milling has been successfully applied to HPF samples [27,28]. FIB-milling lacks typical CEMOVIS artefacts such as knife marks, compression, crevassing, and chatter [29], and it can produce thicker specimens that include more of the cellular structure.

Currently, the major limitations of FIB-milling are the availability of FIB–SEM instruments and expertise. The low throughput of this preparative technique for cryo-tomography makes it a challenge to obtain large datasets, especially important for STA. When applied to HPF specimens, this technique presents additional practical difficulties. Nevertheless, cryo-tomography of FIB-milled specimens is giving spectacular insights into molecular structures *in situ*, and there is hope that FIB-milling will become more widely accessible in the future [12,13,30].

## Data collection

To acquire a tomogram, 2D projections are collected at defined tilt increments [31]. Acquiring tilt series of frozen specimens has posed many longstanding technical challenges [32], as fractionation of electron dose over the entire tilt range ultimately leads to low signal-to-noise ratios (SNR), particularly at higher tilts where the sample is thicker. This makes tracking and focusing of cryo-samples challenging, particularly when using microscopes with unstable specimen stages. Strict microscope calibrations and alignments are essential for collecting a successful tilt series, especially at higher magnifications.

The latest generation of microscopes and detectors, together with improvements in data collection and software, have realised reliable automation of cryo-tomography at higher magnifications, paving the way for molecular resolution from *in situ* cryo-tomography.

### Tilt series acquisition

A tilt series is defined by the tilt range, increment and order [33]. Due to the slab geometry of the EM grid, the thickness of the specimen increases at higher angles, limiting the tilt range to approximately ±70°, with ±60° being common practice. Tomograms consequently have a ‘missing wedge' of information, manifested as anisotropic resolution in the direction of the electron beam.

The tilt increment is typically a uniform step between 0.5° and 5°. Alternative tilt geometries have been proposed to compensate for the loss of information transfer resulting from increased thickness [34], although this is not common practice. The current consensus is to maximise the high-resolution information obtained from lower angle tilts, where the specimen is thinnest. Since electron damage deteriorates high-frequency information faster than low frequencies [35], it is important to acquire low tilt angles earlier in the tilt series so that high-frequency information is preserved [36].

**Figure 3.**
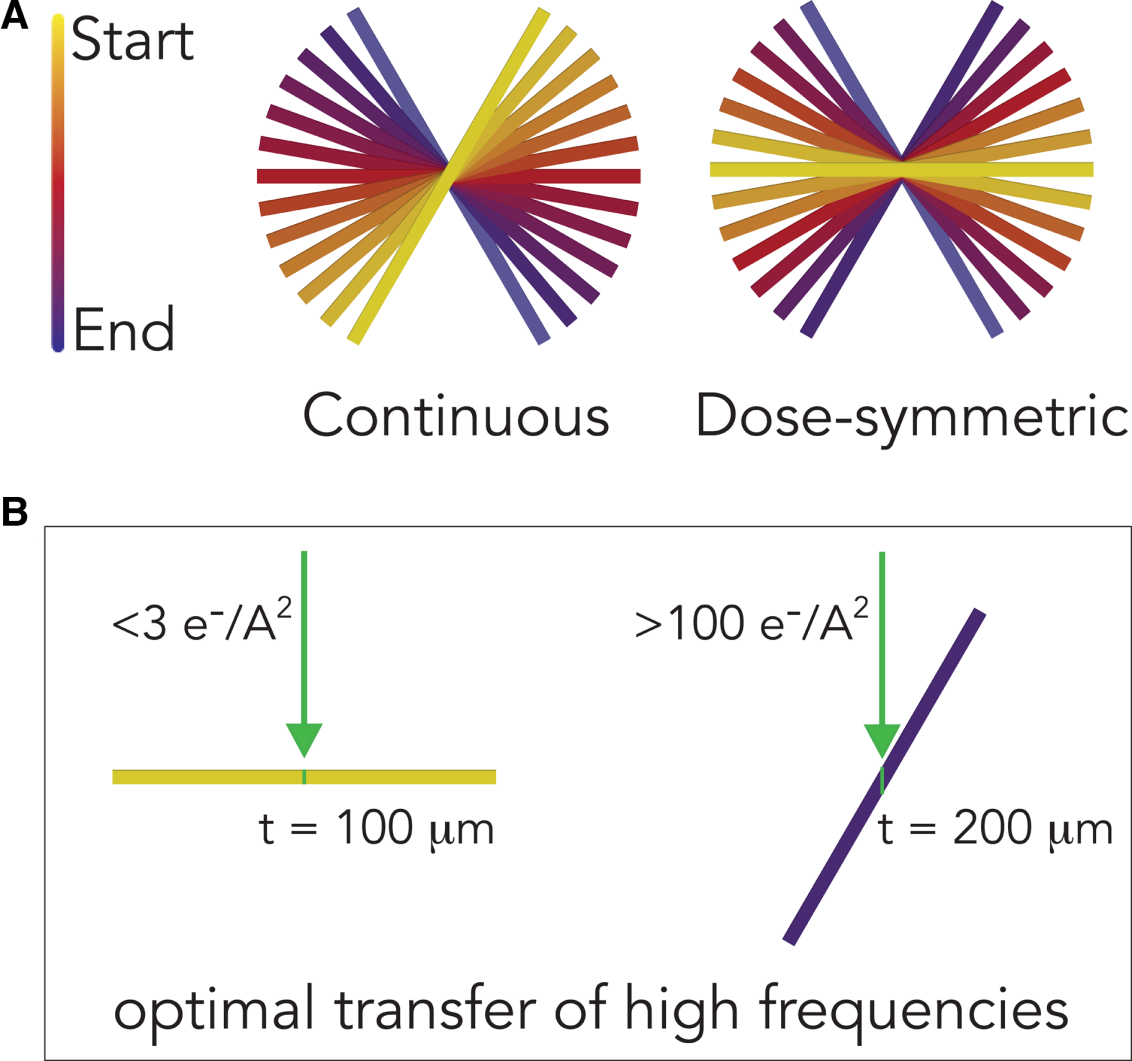
Data collection schemes for cryo-tomography. (**A**) The traditional continuous tilt scheme is shown on the left panel, with data collection starting from high angle and reaching the opposite tilt through sequential increments. The recently implemented dose-symmetric scheme starts at zero, and ‘swings' to increasingly higher tilts. (**B**) A schematic view of how the dose-symmetric scheme transfers high frequencies with optimal efficiency, making it ideal for improving SNR by dose compensation and obtain high resolutions by STA. At low tilts (left panel), high-frequency transfer is highest due to electrons traversing the specimen at its thinnest. Since low tilts are the initial stages of the tilt acquisition, the electron dose accumulated is low, and high-resolution features are less damaged. At high tilts, towards the end of the tomogram collection, high-resolution features are damaged by the beam, and the increased thickness of the tilted specimen is such that the transfer of high-frequency information is weak.

This is achieved with a dose-symmetric tilt scheme, which starts at 0° then acquires alternating positive and negative tilts, and can be currently implemented as a scripted macro within the SerialEM acquisition software [36] (Figure 3). In addition to the optimal use of the thinnest views, the dose-symmetric scheme also avoids the ‘jump-at-zero' problem seen in bidirectional tilt series, whereby large errors are associated with aligning the two halves of the tilt series during tomogram reconstruction [36]. Although modified versions of the bidirectional scheme have been used for subnanometer STA [10,37], the dose-symmetric series has become the *de facto* scheme for cryo-EM tomography and high resolution STA [7,9,11].

### Hardware

New and improved hardware that underlies the ‘resolution revolution' [38] in single-particle cryo-EM also greatly benefits cryo-tomography (Figure 3).

1. Direct electron detector devices (DEDs) have an improved detective quantum efficiency (DQE), which gives better contrast and SNR on low-dose projection images [39,40]. This is particularly evident on higher tilt images, which could become unusable when taken on CCDs, while on DEDs they can show enough features for reliable alignments. Tomographic reconstructions also display better contrast and are therefore more interpretable. Moreover, fractionation of dose across multiple frames (‘movie mode') allows motion correction of individual tilts, limiting the effect of specimen drift, which can be more pronounced upon tilting [41–43]. Thanks to the adaptation of dose-compensation schemes developed for DED in single-particle cryo-EM [35] (see image processing section), total electron doses exceeding 100 electrons/A^2^ have been used to obtain cryo-tomograms with higher SNR, in the knowledge that inflicted damage at high resolutions can be later removed as a post-processing step (see, for example, [11]).
2. Development of the latest generation of electron microscopes has played an important role in the increased quality and throughput of cryo-tomography: (i) Parallel illumination can be easily achieved on the newest lens systems, (ii) stages have become more stable, and (iii) multispecimen holders and autoloaders allow faster screening of grids prior to data collection. Together with improvements in the acquisition software, reliable automated data collection at higher magnification for prolonged periods is now accessible [36,44].
3. Phase plates allow imaging specimens at focus with greatly increased contrast [45]. In cryo-tomography, where objects are pleomorphic and surrounded by crowded environments, phase contrast imaging can be a fundamental aid in the interpretation of the reconstruction [13]. While the use of phase contrast is not standard for cryo-tomography, particularly for FIB-milled specimens, current and future developments will lead to more phase contrast imaging.

## Data processing

After collection of a cryo-tilt series, the individual tilt images must be aligned, their signal restored, and a 3D tomogram must be reconstructed. Interpretation of the cryo-tomogram often requires further processing, including filtering and, if applicable, STA. Below, we summarise the major processing steps, and highlight where standard protocols have improved since the introduction of DEDs.

### Tilt series alignment

Each tilt image is now collected as a series of movie frames (typically 5–10), which are aligned and averaged using the standard motion correction software available for single-particle movie processing. Unlike in typical single-particle workflows, tilt movies should not be dose-compensated using default settings on motion correction software, as dose accumulates on a tilt-by-tilt basis, and each tilt image must be dose-compensated accordingly (see below).

Alignment of tilt images has traditionally been based on gold fiducials, as relying on cross-correlation between the inherent signal of the biological sample at subsequent tilts (fiducial-less) could be problematic due to low SNR. Thanks to the better signal achieved with DEDs, together with the tendency to acquire higher doses per tilt, fiducial-less alignments have recently been shown to perform as well as fiducial-based ones [46].

### Contrast transfer function determination and correction

TEM images are modulated by a contrast transfer function (CTF), which describes an oscillation between positive and negative contrast as a function of spatial frequency (appearing as black and white rings in images power spectra, or Thon rings) [47]. To interpret images at high spatial frequencies, the CTF must be accurately determined based on Thon ring oscillations, and the images must subsequently be corrected. Dealing with CTF in cryo-tomography has traditionally been difficult because of the low SNR in individual tilts, with one or two Thon rings visible in their power spectrum, often none at high tilts [48,49]. Initial attempts at CTF estimation and correction sufficed to restore signal to intermediate resolutions [49]. With DEDs, visible thon rings can exceed subnanometer resolution, although for thicker specimens at high tilt CTF estimation tends to be less accurate. Detection of the CTF can be performed as an average of the whole tilt image, although this might introduce errors and alternative approaches such as periodogram averaging have been proposed [48].

**Figure 4.**
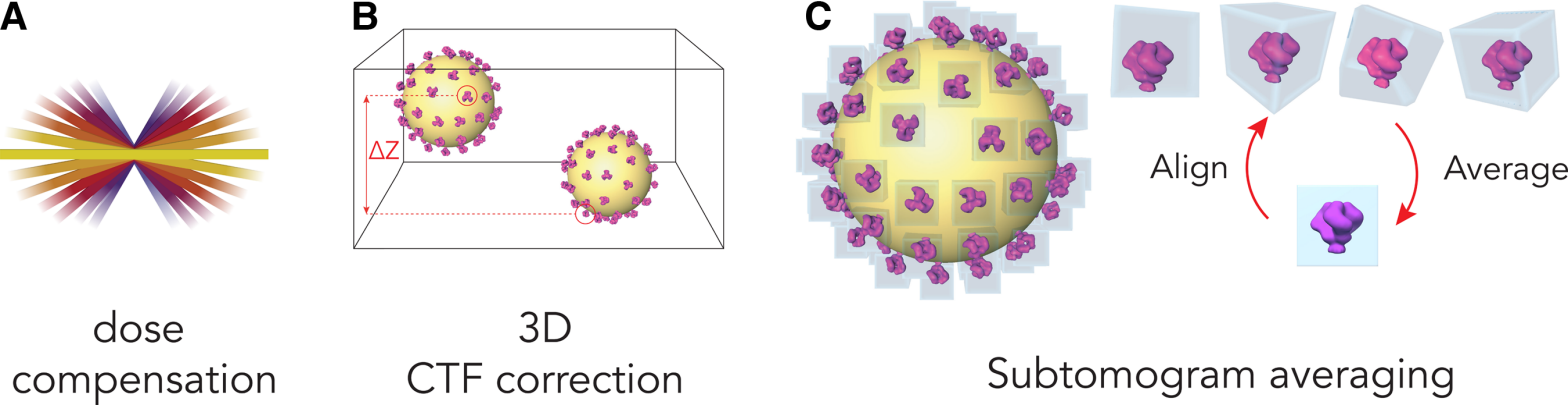
A summary of recent data processing implementations. (**A**) Dose compensation coupled with a dose-symmetric acquisition scheme maximises SNR of the reconstructed tomogram. In each tilt image, high frequencies are attenuated to compensate for the effect of accumulated dose. High-angle tilts accumulating more dose are low pass-filtered to a greater extent than low-angle tilts. (**B**) In the tomogram, each voxel can be assigned a height-adjusted defocus value, allowing CTF correction on a voxel-by-voxel basis. This is particularly advantageous since specimens for tomography are often thicker than those used in single-particle experiments, therefore exhibiting a defocus gradient along the sample depth. (**C**) STA: when multiple copies of the same object are present, they can be aligned and averaged to obtain an isotropic, higher resolution structure.

CTF correction can then be performed taking into account the geometry of each tilt on a tile-by-tile basis [49,50]. Recent approaches perform a 3D or per-particle CTF correction, where each voxel in the tomogram is corrected for the right defocus value by considering its depth in the sample. This can be applied during tomogram reconstruction [51,52] or during STA [53,54] (Figure 4).

### Dose compensation

Dose-compensation protocols across movie frames have been developed for single-particle EM since the advent of DEDs. A dose-dependent attenuation factor is applied to decrease the contribution of low SNR high-frequency information [35]. The same concept can be transferred to a tomography tilt series, where dose accumulates at successive tilts. Coupled with the dose-symmetric tilt scheme, dose-compensation effectively removes high-frequency data that has been degraded by electron damage, increased specimen thickness, and less precise CTF determination (Figure 4), yielding tomograms with an appreciable increase in SNR [11].

### 3D reconstruction and visualisation

Processed tilts are combined in space according to their determined geometry usually by weighted backprojection. SIRT (Simultaneous iterative reconstruction technique)-based algorithms, which tend to increase the SNR, can also be applied and are generally preferred for the visualisation of pleomorphic structures. Other filters might also be applied, for example non-linear anisotropic diffusion, which improves the contrast while preserving edge features in 3D [55].

Segmentation of features, such as cytoskeleton and membranes, is often done to aid interpretation of the reconstructed volume, and is particularly useful for interpreting distorted features due to the missing wedge. Even though some degree of automation has been implemented, this process often involves a great deal of manual tracing and subjective choices. Many segmentation suites are now available that aim to minimise user input (for example [56–59]). These are welcome implementations, increasing the objectivity and throughput of segmentation, therefore keeping up with the increased throughput of data collection.

### Subtomogram averaging

A cryo-tomogram containing multiple copies of the same object in multiple orientations can be processed by STA (Figure 4). Subtomograms containing the features of interest are extracted and undergo iterations of alignment and averaging, enhancing the SNR. STA has many advantages: (1) assuming particle orientations are distributed across a full angular range, STA also compensates for the missing wedge of information inherent to tomographic reconstructions, thereby providing an isotropic view of the repeating object [33]. (2) Particle extraction from a 3D volume rather than a projection image means overlapping features along the depth of the specimen are excluded, allowing structural characterisation of specimens in crowded environments. (3) Positions and orientations of subtomograms can be used to determine the relationship between the molecules of interest and the surrounding environment [33]. Many STA software packages now exist for the processing of larger and better quality datasets, making high-resolution STA more accessible [60–66].

**Figure 5.**
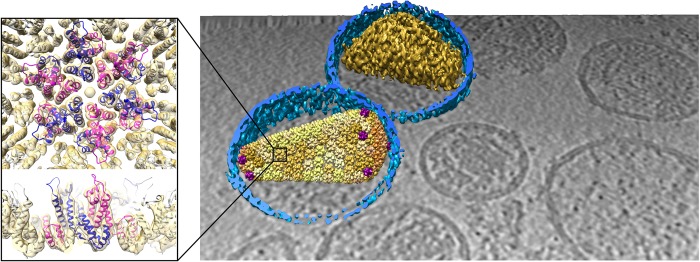
An example of structural determination by cryo-tomography and STA. Mature HIV viruses have irregularly shaped cores that are formed through assembly of hexameric capsid proteins, with pentameric defects. Due to their pleiomorphic nature, other structural biology techniques have failed at determining the native capsid structure and assembly. Here, Mattei et al. [6] were able to obtain reconstructions of hexamers and pentamers to a resolution sufficient to fit atomic models, revealing conformational variations within individual virions. The left panel shows a 6.8 Å structure of the hexamer, with fitted capsid proteins. The right panel shows the superimposition of segmented density (membranes in blue, furthermost core in gold), with the average hexamers and pentamers placed in the tomogram according to their aligned positions and orientations (pentamers in purple, hexamers in shades of yellow depending on cross-correlation scores, darker is higher cross-correlation). Hexamer and fitted model: EMDB 3465, PDB 5mcx. Raw tomogram and capsid co-ordinates courtesy of Simone Mattei and John Briggs.

**Figure 6.**
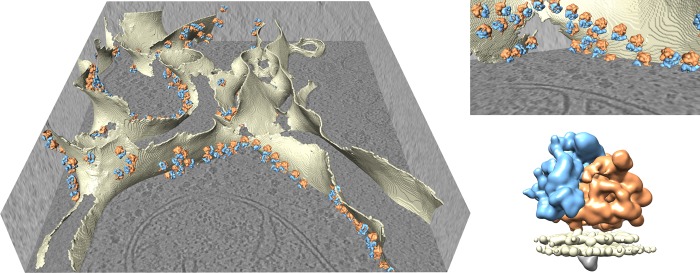
An example of structural determination by cryo-tomography and STA within FIB-milled *Chlamydomonas reinhardtii* cell [**14**]. The left and top right panels show a greyscale slice through a FIB-milled tomogram with ER membranes rendered in solid beige. The 19.4 Å subtomogram average of the ER-associated ribosome (EMDB 4145, bottom right panel: large ribosome subunit, orange; small subunit, blue; membrane, beige) is back-plotted according to aligned positions and orientations, demonstrating the *in situ* arrangement of ER-associated ribosomes. The resolution was sufficient for the authors to deduce the domain organisation of this complex. Raw tomogram and ribosome co-ordinates courtesy of Stefan Pfeffer and Friedrich Förster.

The recent advances in sample preparation, data collection, and processing have culminated in the first near-atomic resolution structure solved by STA [11,51]. This was achieved on immature HIV capsids with regular lattices and symmetry. Elsewhere, subnanometer resolutions have also been achieved for other viral and bacterial structures [6,7,9] (Figure 5), *in vitro* reconstituted coat complexes [8], and even sparser irregularly arranged particles such as ribosomes [10,67,68]. Recently, structures from within whole cells prepared by FIB–SEM have started to emerge [12–14] (Figure 6), unlocking the exciting perspective of visualising biological molecules within cells at molecular detail.

## Conclusions and future perspectives

Here, we have provided a review of the steps involved in CET, from sample preparation to data processing and interpretation. We have focussed, in particular, on current improvements that contribute to spectacular insights into biological samples that were not available for structural investigation until very recently.

Cryo-EM-based techniques are on a fast-rising trajectory, as demonstrated by trends of map depositions on the EMDB [69], and the steady improvements in resolutions that are achieved. Further improvements can be expected for the near future, which will greatly benefit cryo-tomography.

A first expectation is that sample preparation, in particular, FIB-milling of cells and tissues, will become higher-throughput and more accessible to both structural and cell biologists.

More widespread and democratic access to sample screening and data collection will also be important for the growth of cryo-tomography.

There are great margins for improvements also for hardware (e.g. faster and higher-DQE DEDs, easier-to-use phase-plates, faster and more stable specimen stages), and software (e.g. alternative or more sensitive CTF determination algorithms, distortion-corrected tomographic reconstruction, and user-friendly STA programmes).

These improvements will lead to larger datasets with a parallel increase in the quality of reconstructions, realising molecular resolution of decreasingly abundant and large molecules within crowded cellular environments.

## Abbreviations

CEMOVIS: cryo-EM of vitrified specimens
CET: cryo-electron tomography
CTF: contrast transfer function
DEDs: direct electron detector devices
DQE: detective quantum efficiency
EM: electron microscopy
FIB: focused ion beam
HPF: high-pressure freezing
SEM: scanning electron microscopy
SNR: signal-to-noise ratios
STA: subtomogram averaging
